# Changes in Sleep in Children and Adults with Cystic Fibrosis and Primary Ciliary Dyskinesia over Time and after CFTR Modulator Therapy

**DOI:** 10.3390/jcm12247612

**Published:** 2023-12-11

**Authors:** Malena Cohen-Cymberknoh, Maya Lehavi, Alex Gileles-Hillel, Ohad Atia, Oded Breuer, Joel Reiter

**Affiliations:** 1 Pediatric Pulmonary Unit, Cystic Fibrosis Center and Sleep Unit, Hadassah Medical Center, Jerusalem 9112001, Israel; malena@hadassah.org.il (M.C.-C.);; 2 Faculty of Medicine, Hebrew University of Jerusalem, Jerusalem 9112102, Israel; 3 Department of Military Medicine and “Tzameret”, Faculty of Medicine, Hebrew University of Jerusalem, and Medical Corps, Israel Defense Forces, Jerusalem 9190501, Israel; 4 Department of Pediatrics, Shaare Zedek Medical Center, Jerusalem 9103102, Israel

**Keywords:** cystic fibrosis, primary ciliary dyskinesia, sleep, modulator therapy

## Abstract

Cystic fibrosis (CF) and primary ciliary dyskinesia (PCD) are associated with sleep disturbances affecting quality of life (QOL) in both children and adults. However, little is known about the progression of these complaints over time, and the effect of CFTR modulator (CFTRm) therapies. Participants completed sleep quality (SDSC, PSQI) and quality of life questionnaires (PedQL, QOL-BE) as well as the Epworth sleepiness scale (ESS) at baseline and after 4 years. Medical records were reviewed for clinical data and correlations were sought between sleep, QOL, and clinical parameters. A total of 67 patients (33 pediatric), 37 pancreatic insufficient CF (CF-PI), 15 pancreatic sufficient CF (CF-PS), and 15 PCD patients, completed the study. In adults, global sleep quality decreased from 85.8% (76.2–90.5) to 80.9% (71.4–85.7); (*p* = 0.009). Analysis by disease cohort showed a significant deterioration only in the CF-PS group. In adults off CFTRm, sleep quality decreased from 85.7% (78.6–88.2) to 80.9% (71.4–87.3); (*p* = 0.021) and from 85.8% (76.2–92.9) to 76.2% (71.4–85.8); (*p* = 0.078) in people on CFTRm. Changes in sleep quality and changes in QOL over time were strongly associated with each other. In conclusion sleep quality deteriorates over time, correlates with QOL, and is driven primarily by adults and CF-PS patients. CFTRm has a possible effect on sleep initiation; however, results are mixed, and further long-term studies are required.

## 1. Introduction

Cystic fibrosis (CF) and primary ciliary dyskinesia (PCD) share clinical signs and symptoms but differ in other ways [1,2]. Multisystemic diseases, studies in people with CF (pwCF) have shown decreased sleep efficiency and symptoms suggestive of obstructive sleep apnea (OSA), poor sleep quality, and daytime sleepiness, possibly associated with decreased quality of life (QOL) [3]. Sleep quality questionnaires show poor sleep quality in adults [4], and more sleep disturbances in children with PCD [5], compared with controls. In a previous study that compared sleep disorders between pwCF that were pancreatic insufficient (CF-PI), pancreatic sufficient (CF-PS), and PCD patients, we had shown that despite clinical and pathophysiologic differences between the three conditions, sleep disorders were common, similar, and correlated with QOL [3,6].

Little is known about the progression of sleep disturbance complaints over time in these disorders. Furthermore, the availability of CFTR modulator therapies (CFTRm) has led to dramatic improvements in the QOL and the prognosis of pwCF. However, there is only minimal data on the effects of CFTRm on sleep, suggesting a possible worsening of sleep in some, no change, and improvement [7] in others (mostly case reports and series [8,9,10,11,12]).

The objectives of the current study were to analyze changes in sleep complaints over time and to explore the effect of CFTRm therapies. To achieve the latter, we compared changes in sleep complaints between pwCF on and off CFTRm, as well as pwPCD, in whom no new therapies have become available. Furthermore, we aimed to assess the correlation between changes in sleep complaints and changes in clinical disease parameters as well as the effect of these changes on patients’ QOL.

## 2. Materials and Methods

### 2.1. Subjects

People with a confirmed diagnosis of CF or PCD followed at the CF Center at the Hadassah-Hebrew University Medical Center, who participated in our previous study conducted between 2016–2017, were asked to take part in this follow-up study, conducted during 2020–2022 [6]. The study was approved by the local ethics committee and all patients gave informed written consent.

### 2.2. Study Design

All patients completed three validated questionnaires at two time points, on initial enrollment, and 4 years thereafter, a sleep quality questionnaire, a sleepiness scale, and a quality of life questionnaire. Age-specific questionnaires were completed according to the age on initial enrollment, i.e., baseline, to allow for comparison of changes over time. Adults completed the Pittsburg Sleep Quality Index (PSQI), the Epworth Sleepiness Scale (ESS) and the Quality of Life Questionnaire-Bronchiectasis (QOL-B). Children, up to 18 years old at baseline, completed the Sleep Disturbance Scale for Children (SDSC), the modified Epworth Sleepiness Scale (mESS), and the Pediatric Quality of Life Inventory (PedsQL).

Study Questionnaires:

The PSQI estimates sleep quality and disturbances over one month and has been formerly used in CF patients [13,14]. There are seven subdomains analyzed: subjective sleep quality, sleep latency, sleep duration, habitual sleep efficiency, sleep disturbances, use of sleeping medication, and daytime dysfunction. For the global sleep quality score, a value over 5 is consistent with poor sleep quality [15].

The QOL-B is a health-related quality of life (HRQOL) questionnaire. There are several different scales, including symptoms, physical, social, and emotional functioning [16].

The SDSC classifies sleep disorders over the previous 6 months in children. There are six subdomains, disorders of initiating and maintaining sleep, sleep breathing disorders, disorders of arousal, sleep–wake transition disorders, disorders of excessive somnolence, and sleep hyperhidrosis [17]. Though the instrument is a measure of sleep disturbances, it has been used as an estimation of sleep quality in children using the combined global sleep score [18,19].

The PedsQL questionnaire is a measure of health-related QOL with data on the physical, emotional, social, and school functioning covering the previous 4 weeks [20]. Abnormal scores are established as those lower than the standard error of measurement (SEM) [21].

The ESS rates the respondents’ chances of dozing off or falling asleep while engaged in eight different activities. Scores above 10 are indicative of excessive daytime sleepiness, and scores between 5 and 10 are indicative of increased normal range daytime sleepiness [22]. In children, parents, or the children themselves completed the Childhood Adenotonsillectomy Trial (CHAT) modified ESS as described by Paruthi et al. [23].

### 2.3. Clinical Data

Clinical data were collected from patients’ electronic medical records. Data included demographic information, body mass index (BMI), laboratory blood results, measures of lung disease severity, and Pseudomonas infection status (defined by Leeds criteria as: intermittent-pseudomonas positive < 50%; chronic-positive > 50% of the explored months) [24], and number of severe pulmonary exacerbations (PEx), requiring intravenous (IV) antibiotics and/or hospitalization during the 12 months prior to enrolment. In pwCF, the medical records were also reviewed for treatment with modulator therapies and for a diagnosis of CF-related diabetes mellitus (CFRD).

### 2.4. Outcome Measures

The primary outcome measure of the study was the change in global sleep quality over time. Additional outcome measures included correlations between changes in global and domain-specific sleep characteristics and clinical parameters such as the specific disease, i.e., CF-PS, CF-PI, and PCD; lung function; BMI; Pseudomonas infections; PEx; hemoglobin; vitamin D; CFRD, use of caffeine, etc., and the correlations between changes in sleep quality and changes in QOL over time.

### 2.5. Statistical Analysis

Results are shown for current study questionnaires followed by changes from baseline. Quantitative variables are shown as means and standard deviations (SD) in parametric and medians and interquartile range in non-parametric tests. Qualitative variables are shown as frequencies and percentages. As noted, for uniform representation of the results, and to allow multiple comparisons, scores are shown as the percent of total achievable score. To test the association between two categorical variables, the chi-square test, and Fisher’s exact test, were used. For the comparison of quantitative variables, between two independent groups, the non-parametric Mann–Whitney test was used. Comparison of quantitative variables, between three independent groups, was performed with the non-parametric Kruskal–Wallis test, with post hoc tests and the Bonferroni correction of the significance level. The Spearman non-parametric correlation coefficient was calculated to assess the strength of the correlation between two quantitative variables. To analyze the change between two time points, for a quantitative variable, within a study group, the Wilcoxon signed ranks non-parametric test was used. Non-parametric tests were used due to the small sample sizes and the non-normal distribution of some of the variables. All tests applied were two-tailed, and a *p*-value of ≤0.05 was considered statistically significant.

## 3. Results

### 3.1. Patient Characteristics

Patient characteristics are shown in Table 1. Overall, 67 patients (37 CF-PI, 15 CF-PS, and 15 PCD) completed the current study with a mean interval of 4 years from the prior study. There were 34 adults with a median age of 29 (25–38) years. A total of 30 pwCF were on CFTRm therapies: 11 pwCF-PS and 19 pwCF-PI: 6 patients on tezacaftor–ivacaftor, 1 on lumacaftor–ivacaftor, and 23 on elexacaftor–tezacaftor–ivacaftor at the time of the study. Adults had a mild obstructive impairment, normal resting oxygen saturations, and a median BMI of 23.6 kg/m^2^. Chronic Pseudomonas infection was present in 7 (20.6%) and 12 (35.3%) had CFRD. Over the year prior to the participation in the study, 11 (32%) had a severe PEx (hospitalization, IV antibiotics, or both).

A total of 33 patients with a median age of 15 (12–19) completed the pediatric questionnaires. Children had mildly reduced pulmonary function and normal oxygen saturations. Pseudomonas infection was present in 12 (36%) children and 12 (36%) had a severe PEx during the year prior to the study.

### 3.2. Changes in Sleep Quality over Time

Changes in sleep over time are shown in Table 2 and Table 3 for adults and children, respectively. In adults, global sleep quality on the PSQI decreased from 85.8% (76.2–90.5) to 80.9% (71.4–85.7); (*p* = 0.009). Subdomain analysis showed a decrease in the sleep latency domain from 100 (83.3–100) to 83.3% (66.6–100); (*p* = 0.009). In children, there was no statistically significant change in sleep quality.

### 3.3. Changes in Sleep Quality According to Disease

Sleep quality of adults and children decreased significantly only in the CF-PS group, with global sleep quality scores decreasing from 90.5% (76.8–95.7) to 80.95% (71.4–90.7); (*p* = 0.006) (Supplementary Table S5). There were no significant changes over time among the CF-PI or PCD groups. On analysis according to age group, sleep quality decreased in adults with CF-PS from 85.8% (76.4–90.5) to 76.2% (71.4–85.7); (*p* = 0.018), but not in children with CF-PS (95.7% (83.4–97.5) to 90.7% (55.4–92.8); *p* = 0.116) (Table 2 and Table 3).

### 3.4. Changes in Sleep Quality According to CFTRm Therapy

Analysis was performed on 30 pwCF on CFTRm therapy and 37 off CFTRm. Most patients on CFTRm were started on the medication between initial enrollment and follow-up, with several patients changing from one CFTRm to another during the interval. In adults, a decrease in global sleep quality was seen in both patients on and off therapy. Global sleep quality decreased from 85.7% (70.8–92.5) to 80.9% (71.4–87.3); (*p* = 0.021) in patients off CFTRm and from 85.8% (76.2–92.9) to 76.2% (71.4–85.8); (*p* = 0.078) in pwCF on CFTRm (Supplementary Tables S7 and S8). On subdomain analysis, in patients off CFTRm, the sleep latency subdomain decreased from 100% (74.9–100) to 83.3% (66.6–100); (*p* = 0.034) and sleep disturbances subdomain score, from 87.5% (79–91.7) to 79.2% (64.6–86.6); (*p* = 0.019) (Supplementary Tables S7 and S8). In pwCF that were on CFTRm-sleep latency subdomain score changed from 100% (87.5–100) to 83.3% (54.2–100); (*p* = 0.082) however, the sleep disturbances subdomain score increased from 79.2% (70.8–95.8) to 87.5% (67.7–93.7); (*p* = 0.468) (Supplementary Tables S7 and S8). While these changes are only significant in patients off CFTRm, it seems, this is a result of the small groups (see discussion).

There was no significant change in global sleep quality and QOL among children on (N = 13) and off (N = 20) CFTRm. A deterioration in the initiating and maintaining sleep subdomain on the SDSC was only seen in children that were on CFTRm, 89.3% (83.5–94.8) to 83.3% (80–90); (*p* = 0.054) vs. 81% (60.7–92.8) to 77.1% (68–82.8); (*p* = 0.47), in children on and off CFTRm, respectively.

### 3.5. Correlations—With QOL and Lung Functions

Numerous significant correlations were found between changes in sleep quality questionnaire subdomains and changes in QOL domains in the total group, among children and adults (Tables S1, S3 and S6). Patients who experienced deterioration in certain sleep quality domains also demonstrated a deterioration in QOL domains. There was a significant positive correlation between changes in global sleep quality and changes in total QOL among the total group (correlation coefficient—0.301, *p* = 0.017). We found a correlation between changes in sleepiness represented by the Epworth questionnaire and total QOL (correlation coefficient—0.323 *p* = 0.010). A negative change in the quality of sleep subdomain led to a similar change in QOL (correlation coefficient—0.301; *p* = 0.017). No significant correlations were found between the FEV1 and change in FEV1 over time and changes in sleep quality scores (Supplementary Tables S2 and S4).

## 4. Discussion

Both CF and PCD are systemic diseases in which disturbed or poor quality of sleep have been shown [6]. To the best of our knowledge, this is the first study to explore the dynamics of these complaints over time and compare between pwCF-PI, pwCF-PS, and pwPCD groups. In addition, these data allowed us to examine the differences and possible effects of CFTRm on sleep. In this study, we show that general sleep quality and sleep latency deteriorate over time in adults and that this correlates with QOL. This is mostly driven by pwCF-PS. There appear to be no changes over time in children on general or subdomain analysis of their sleep disturbances. In this study, comparing changes over time in pwCF on and off CFTRm revealed no clear effect on patients’ sleep.

Poor sleep quality on the PSQI questionnaire has been described in adults with CF, in several prior studies. Our initial study showed poor sleep quality in 26.5% of adults with CF [6]. Jankelowitz compared 20 pwCF with healthy individuals, showing higher PSQI scores, which are correlated with objective findings, and a higher fragmentation index in actigraphy [14]. Bouka showed increased ESS and PSQI scores that correlated with lower health-related QOL scores [25]. We found numerous significant correlations between changes over time in the quality of sleep and QOL consistent with prior studies, showing a strong correlation between patients’ quality of sleep and QOL [6]. Suboptimal sleep quality affects the physical capacity of the patients, their emotional and social functioning, and in children, their performance in school [6].

In the pediatric population, evidence of disturbed sleep is not as clear. Vandeleur assessed subjective sleep quality using the SDSC in CF and found higher total scores suggestive of worse sleep quality, compared with controls. The SDSC scores were correlated with disease severity [26]. In contrast, Santamaria found lower total SDSC scores and several subdomain scores in children with PCD compared with controls [5]. The sleep breathing domain demonstrated a higher score in the pwPCD [5]. Oktem utilized the PSQI and found higher scores among pwPCD compared to healthy subjects, with 11 of 29 patients reporting “poor sleep” compared with only 1 control individual [4]. Our previous study, which included pediatric pwCF-PI, pwCF-PS, and pwPCD, demonstrated SDSC global quality suggestive of disturbed sleep in 15.4% of the children [6].

The deterioration in global sleep quality in adults could be partially attributed to normal aging as evidenced by the lack of deterioration in children and adolescents [27]. A meta-analysis that included 3577 subjects showed a decrease in sleep time, worsening sleep efficiency, and an increase in sleep latency with aging in healthy adults [27]. Nevertheless, our population is relatively young, and therefore, age as a factor might be less influential. Another possible explanation may be the natural course of the diseases, which frequently implies gradual deterioration. However, we found no clear correlation between sleep quality and pulmonary functions. The contribution of pwCF-PS to the deterioration of the global sleep quality is unclear, there was no significant difference in clinical parameters between the diseases in the current study, and no difference in sleep parameters in our previous study [6]. Further investigation is needed to explain this result.

The morbidity and mortality of pwCF have seen dramatic changes in recent years due to modulator therapies [28]. It has been suggested that improvement in QOL scores reflects positive changes in many aspects of the patients’ lives [29,30]. However, the effect of these medications on mental health and sleep is not clear. Spoletini described 19 adult pwCF taking Trikafta with anxiety, bad moods, impaired attention, and insomnia [10]. A recent publication by Heo described six pwCF who reported mental status changes after initiation of Trikafta [12]. In a study of 100 pwCF, following Trikafta initiation 22 pwCF had changed their psychiatric medication regimen, 23 participants reported troubled sleep and 5 had a new diagnosis of mental health disorder [31]. Furthermore, improvement in nutritional status has been suggested as a contributor to increased rates of OSA [32].

In the present study, although there are relatively small groups, being on CFTRm had no influence on global sleep quality. Nevertheless, CFTRm use was associated with difficulties falling asleep seen on the initiating and maintaining sleep subdomain scores on the SDSC questionnaire in children, and the PSQI sleep latency domain in adults (the latter, not statistically significant). We hypothesize that the lack of effect seen in pwCF on CFTRm may stem from different effects the medications have on different aspects of sleep—on the one hand, these medications improve pwCF wellbeing and respiratory health, thereby having a positive effect on their sleep, while, on the other hand, having a direct negative effect on pwCF psychologic wellbeing [10] and sleep initiation.

Several limitations should be mentioned. The use of subjective measurements of sleep may lead to reporting bias. Second, the small sample size of the patients’ groups may have impaired the detection of small differences among the subgroups. Due to the small subgroups on CFTRm, the effect of specific medications could not be assessed separately nor could the length of time on the medication. To compare data with the previous study, following a 4-year gap, we asked the participants to complete identical questionnaires to the ones completed 4 years before. Therefore, some pediatric patients completed questionnaires that were not strictly age-compatible. Despite these limitations, the current study has several strengths. It addresses differences in sleep quality over time in pwCF-PI and pwCF-PS separately, a subject that has not been previously studied in the context of sleep, to the best of our knowledge. Moreover, the study adds information regarding the effect of CFTRm therapies on the quality of sleep in different age groups emphasizing the need for additional studies to clarify this.

## 5. Conclusions

In conclusion, a deterioration in global sleep quality was demonstrated among adults with either CF-PI, CF-PS, or PCD, over four years that elapsed from our initial analysis. CFTRm therapies may have an impact on sleep initiation. Additional studies are needed to clarify the reasons for the changes we observed and to ascertain the effects of long-term CFTRm therapy in a larger cohort of pwCF.

## Figures and Tables

**Table 1:** Patient characteristics, total and disease specific.

	Total	CF-PI	CF-PS	PCD	*p* Value *
**Adults**, *n* (female)	34 (18)	19 (10)	8 (4)	7 (4)	
Age, years	29 (25–38)	29 (25–38)	31(25–47)	27 (27–37)	0.972
Oxygen saturation, %	98 (96–99)	98 (96–99)	98 (96.5–100)	97 (95–97.5)	0.181
FEV1, % predicted	73 (61–85)	73 (63–83)	66.5(56–87)	73 (53.5–96.5)	0.852
BMI, kg/m^2^	23.6 (21–25.4)	23.5 (21–24)	23.4 (19.5–26.5)	27.9 (23.1–31.1)	0.166
Pseudomonas infection-chronic	7	3	2	2	0.652
** Patients hospitalized	7	2	2	3	0.042
** Patients on IV antibiotics	10	5	3	2	0.655
Diabetes	12	10	2	0	0.056
Vitamin D, ng/mL	29 (23–43)	36.5 (23–47)	27 (21–28)		0.302
Hemoglobin, g/dL	13.7 (12.8–15.1)	13.5 (12.5–15.1)	13.8 (13.6–14)	14.8 (13.3–15.2)	0.761
**Children** ***, *n* (female)	33 (17)	18 (10)	7 (4)	8 (3)	
Age, years	15 (12–19)	16 (12–19)	16 (12–20)	13 (11–15.5)	0.333
Oxygen saturation, %	98 (97–100)	99 (97–100)	98 (97–99)	97 (97–98)	0.515
FEV1, % predicted	90 (79–99)	89 (79.5–102.5)	92 (88–96)	89 (68–93)	0.447
BMI percentile, %	34 (18–56)	29.5 (18.5–37.5)	45 (17–72)	53 (3–87)	0.397
Pseudomonas infection-chronic	3	3	0	0	0.538
** Patients hospitalized	7	4	0	3	0.131
** Patients on IV antibiotics	9	6	1	2	0.865
Diabetes	10	8	2	0	0.1
Vitamin D, ng/mL	31.5 (22.5–37.5)	33 (22.5–39.5)	31.5 (29–38)		0.305
Hemoglobin, g/dL	13.9 (13.3–14.7)	13.8 (13.3–14.4)	14.0 (13.5–14.7)	15.1 (13.8–15.7)	0.175

Results shown as median and interquartile range deviation or number of patients. * *p* value represents the results of a comparison between CF PI, CF PS, and PCD; ** over 12 months, prior to enrolment; results shown as number of patients or median and interquartile range (IQR). *** Baseline according to age at enrolment. Abbreviations—CF: cystic fibrosis, PI: pancreatic insufficient, PS: pancreatic sufficient, PCD: primary ciliary dyskinesia, FEV1: forced expiratory volume in 1 s, BMI: body mass index, IV: intravenous.

**Table 2:** Changes in sleep, sleepiness, and quality of life over time in adult patients.

	Total N = 34		* *p* Value	CF-PI N = 19		CF-PS N = 8		PCD N = 7		** *p* Value (PI, PS, PCD)
PSQI	Baseline	Follow Up		Baseline	Follow Up	Baseline	Follow Up	Baseline	Follow Up	Follow Up
Sleep quality, global	85.8 (76.2–90.5)	80.9 (71.4–85.7)	**0.009**	85.8 (76.2–90.5)	85.7 (71.4–85.8)	85.75 (76.4–90.5)	76.2 (71.4–85.7)	85.8 (76.2–95.2)	80.9 (71.4–88.9)	0.294, **0.018**, 0.063
Sleep latency	100 (83.3–100)	83.3 (66–100)	**0.009**	100 (83.3–100)	83.3 (62.45–100)	100 (87.3–100)	91.7 (70.78–100)	100 (50–100)	66.6 (50–100)	**0.041**, 0.461, 0.144
Sleep disturbances	85.4 (75–95.7)	83.3 (66.6–91.6)	0.156	83.3 (75–95.8)	85.7 (75–95.8)	89.6 (77.1–95.8)	77.1 (60.4–90.6)	87.5 (58.3–91.7)	71.4 (62.5–83.3)	0.826, 0.233, 0.128
Subjective sleep	100 (66.6–100)	100 (66.6–100)	0.629	100 (66.6–100)	100 (66.6–100)	87.5 (66.2–100)	100 (100–66.6)	100 (66.6–100)	100 (66.6–100)	0.765, 0.752, 0.414
Sleep duration	6.5 (6–7)	6 (5.5–7)	0.26	6.5 (6–7)	6 (5.5–7.5)	6.5 (6–7)	6.3 (6–7)	6.5 (5.5–7.5)	6.5 (5–7.5)	0.49, 0.34, 0.71
**ESS**										
ESS	83.3 (75–91.7)	83.3 (66.6–100)	0.206	75.8 (69.8–84.1)	83.3 (75–87.5)	93.75 (87.5–100)	70.8 (62.5–75)	86.6 (79.1–100)	83.3 (70.8–95.8)	0.196, **0.021**, 0.398
**QOL-B**										
QOL global score	73.6 (63.0–83.8)	75.7 (63–83.8)	0.417	76.2 (63.5–81.3)	80.3 (74.6–89.2)	68.2 (51.9–82.4)	66.3 (53.2–88.1)	67.8 (48.1–88.1)	60.4 (52.3–76.6)	0.365, 0.237, 0.345

* *p* value represents the results of the comparison of baseline and current results of the total group; results are shown as (median and interquartile range). ** *p* value represents the results of the comparison of baseline and current results of CF PI, CF PS, and PCD; results as median and (interquartile range). Results are shown as the percent of the total possible score, where 100% is always the best, i.e., no disorder. Sleep duration is shown in hours. Abbreviations—CF: cystic fibrosis, PI: pancreatic insufficient, PS: pancreatic sufficient, PCD: primary ciliary dyskinesia, ESS: Epworth sleepiness scale, PSQI: Pittsburgh Sleep Quality Index, QOL: quality of life, QOL-B: Quality of Life Bronchiectasis Pills subdomain is not in the table, only one patient used sleeping pills.

**Table 3:** Changes in sleep, sleepiness, and quality of life over time in children.

	Total N = 33		* *p* Value	CF-PI N = 18		CF-PS N = 7		PCD N = 8		** *p* Value (PI, PS, PCD)
SDSC	Baseline	Follow Up		Baseline	Follow Up	Baseline	Follow Up	Baseline	Follow Up	
Sleep quality, global	89.5 (75.4–94.6)	90.7 (81.2–96.1)	0.25	85.6 (82.2–92.1)	90.7 (75.9–94.9)	95.7 (83.4–97.5)	90.7 (55.4–92.8)	94.2 (71.4–96.1)	79.2 (75.4–95.3)	0.943, 0.116, 0.498
Initiating and maintaining sleep	87.4 (75–93.0)	82.8 (74.3–87.8)	0.16	83.9 (71.4–92.9)	82.8 (74.3–88.5)	91 (72.3–94.8)	83.3(80–88.6)	91.1 (68.3–92.8)	74.2 (62.9–82.8)	0.795, 0.345, 0.128
Sleep/wake transition disorder	95.8 (83.3–100)	96.6 (90–100)	0.91	87.5 (83.3–95.9)	96.6 (91.7–100)	100 (87.5–100)	96 (76–100)	100 (72.9–100)	93.3 (86.6–100)	0.187, 0.080, 0.500
Sleep breathing disorder	100 (83.3–100)	100 (73.3–100)	0.47	100 (83.3–100)	100 (93.3–100)	100 (76.8–100)	100 (66.6–100)	91.7 (68.2–100)	53.3 (33.3–100)	0.310, 0.285, 0.141
Hyperhidrosis disorder	100 (75–100)	100 (70–100)	0.19	100 (81.3–100)	100 (90–100)	100 (81.3–100)	100 (70–100)	87.5 (65.6–100)	70 (60–100)	0.916, 0.276, 0.066
Arousal disorder	100 (100–100)	100 (100–100)	0.25	100 (100–100)	100 (96.7–100)	100 (81.2–100)	100 (93.3–100)	100 (100–100)	100 (100–100)	0.336, 1.000, 0.317
Excessive Somnolence disorder	85 (68–100)	88 (72–100)	0.59	75 (65–87.5)	88 (76–100)	100 (87.5–100)	84 (72–88)	90 (72.3–100)	92 (64–100)	0.044, 0.343, 0.248
**ESS**										
ESS	83.3 (70–87.5)	90 (73.3–93.3)	0.45	86.6 (68.7–95.8)	90 (81.1–93.3)	80.8 (68.9–89.9)	90 (70–93.3)	79.2 (67.5–86.5)	71.7 (55.0–88.3)	0.236, 0.345, 0.398
**PedsOL**										
QOL global score	83.3 (70.9–93.5)	79.9 (66–90.2)	0.51	83.7 (76.1–93.9)	86.9 (69.6–95.1)	63.2 (52.8–93.5)	75 (75–84.7)	83.3 (67.8–87.8)	73.9 (57.1–80.1)	0.538, 0.499, 0.161

* *p* value represents the results of the comparison of baseline and current results of the total group; results are shown as (median and interquartile range). ** *p* value represents the results of the comparison of baseline and current results of CF PI, CF PS, and PCD; results as median and (interquartile range). Results are shown as the percent of the total possible score, where 100% is always the best, i.e., no disorder. Abbreviations—CF: cystic fibrosis, PI: pancreatic insufficient, PS: pancreatic sufficient, PCD: primary ciliary dyskinesia, ESS: Epworth sleepiness scale, SDSC: The Sleep Disturbance Scale for Children, QOL: quality of life, PedsQL: Pediatric Quality of Life Inventory.

## Data Availability

The data presented in this study are available in the article and supplementary material.

## Supplementary Materials

The following supporting information can be downloaded at: https://www.mdpi.com/article/10.3390/jcm12247612/s1, Supplementary Table S1: Correlation between changes in sleep, sleepiness, and quality of life over time in adults, Supplementary Table S2: Correlation between changes in sleep, sleepiness, QOL and FEV1, FEV1 change in adults, Supplementary Table S3: Correlation between changes in sleep, sleepiness, and quality of life over time in pediatric patients, Supplementary Table S4: Correlation between changes in sleep, sleepiness, QOL and FEV1, FEV1 change in pediatric patients, Supplementary Table S5: Changes over time in sleep, sleepiness and QOL characteristics in total group, Supplementary Table S6: Correlation between changes in sleep, sleepiness and QOL in total group, Supplementary Table S7: Changes in sleep, sleepiness and QOL in patients off modulators, Supplementary Table S8: Changes in sleep, sleepiness and QOL in patients on modulators.

## References

[1] Simanovsky N., Cohen-Cymberknoh M., Shoseyov D., Gileles-Hillel A., Wilschanski M., Kerem E., Hiller N. (2013). Differences in the pattern of structural abnormalities on CT scan in patients with cystic fibrosis and pancreatic sufficiency or insufficiency. Chest.

[2] Cohen-Cymberknoh M., Simanovsky N., Hiller N., Hillel A.G., Shoseyov D., Kerem E. (2014). Differences in disease expression between primary ciliary dyskinesia and cystic fibrosis with and without pancreatic insufficiency. Chest.

[3] Reiter J., Gileles-Hillel A., Cohen-Cymberknoh M., Rosen D., Kerem E., Gozal D., Forno E. (2020). Sleep disorders in cystic fibrosis: A systematic review and meta-analysis. Sleep Med. Rev..

[4] Oktem S., Karadag B., Erdem E., Gokdemir Y., Karakoc F., Dagli E., Ersu R. (2013). Sleep disordered breathing in patients with primary ciliary dyskinesia. Pediatr. Pulmonol..

[5] Santamaria F., Esposito M., Montella S., Cantone E., Mollica C., De Stefano S., Mirra V., Carotenuto M. (2014). Sleep disordered breathing and airway disease in primary ciliary dyskinesia. Respirology.

[6] Cohen-Cymberknoh M., Atia O., Gileles-Hillel A., Kerem E., Reiter J. (2019). Sleep disorders in patients with primary ciliary dyskinesia, cystic fibrosis with and without pancreatic insufficiency. Respir. Med..

[7] Meltzer L.J., Gross J.E. Characterization of sleep in emerging adults with cystic fibrosis on elexacaftor/tezacaftor/ivacaftor. J. Cyst. Fibros..

[8] Tindell W., Su A., Oros S.M., Rayapati A.O., Rakesh G. (2020). Trikafta and Psychopathology in Cystic Fibrosis: A Case Report. Psychosomatics.

[9] McKinzie C.J., Goralski J.L., Noah T.L., Retsch-Bogart G.Z., Prieur M.B. (2017). Worsening anxiety and depression after initiation of lumacaftor/ivacaftor combination therapy in adolescent females with cystic fibrosis. J. Cyst. Fibros..

[10] Spoletini G., Gillgrass L., Pollard K., Shaw N., Williams E., Etherington C., Clifton I., Peckham D. (2022). Dose adjustments of Elexacaftor/Tezacaftor/Ivacaftor in response to mental health side effects in adults with cystic fibrosis. J. Cyst. Fibros..

[11] Dagenais R.V.E., Su V.C.H., Quon B.S. (2020). Real-World Safety of CFTR Modulators in the Treatment of Cystic Fibrosis: A Systematic Review. J. Clin. Med..

[12] Heo S., Young D.C., Safirstein J., Bourque B., Antell M.H., Diloreto S., Rotolo S.M. (2022). Mental status changes during elexacaftor/tezacaftor / ivacaftor therapy. J. Cyst. Fibros..

[13] Milross M.A., Piper A.J., Norman M., Dobbin C.J., Grunstein R.R., Sullivan C.E., Bye P.T. (2002). Subjective sleep quality in cystic fibrosis. Sleep. Med..

[14] Jankelowitz L., Reid K.J., Wolfe L., Cullina J., Zee P.C., Jain M. (2005). Cystic fibrosis patients have poor sleep quality despite normal sleep latency and efficiency. Chest.

[15] Buysse D.J., Reynolds C.F., Monk T.H., Berman S.R., Kupfer D.J. (1989). The Pittsburgh Sleep Quality Index: A new instrument for psychiatric practice and research. Psychiatry Res..

[16] Olveira C., Olveira G., Espildora F., Giron R.M., Muñoz G., Quittner A.L., Martinez-Garcia M.A. (2014). Validation of a Quality of Life Questionnaire for Bronchiectasis: Psychometric analyses of the Spanish QOL-B-V3.0. Qual. Life Res..

[17] Bruni O., Ottaviano S., Guidetti V., Romoli M., Innocenzi M., Cortesi F., Giannotti F. (1996). The Sleep Disturbance Scale for Children (SDSC). Construction and validation of an instrument to evaluate sleep disturbances in childhood and adolescence. J. Sleep. Res..

[18] Rönnlund H., Elovainio M., Virtanen I., Matomäki J., Lapinleimu H. (2016). Poor Parental Sleep and the Reported Sleep Quality of Their Children. Pediatrics.

[19] Roeser K., Eichholz R., Schwerdtle B., Schlarb A.A., Kübler A. (2012). Relationship of sleep quality and health-related quality of life in adolescents according to self- and proxy ratings: A questionnaire survey. Front. Psychiatry.

[20] Varni J.W., Seid M., Rode C.A. (1999). The PedsQL: Measurement model for the pediatric quality of life inventory. Med. Care.

[21] Varni J.W., Burwinkle T.M., Seid M., Skarr D. (2003). The PedsQL 4.0 as a pediatric population health measure: Feasibility, reliability, and validity. Ambul. Pediatr..

[22] Johns M., Hocking B. (1997). Daytime sleepiness and sleep habits of Australian workers. Sleep.

[23] Paruthi S., Buchanan P., Weng J., Chervin R.D., Mitchell R.B., Dore-Stites D., Sadhwani A., Katz E.S., Bent J., Rosen C.L. (2016). Effect of Adenotonsillectomy on Parent-Reported Sleepiness in Children with Obstructive Sleep Apnea. Sleep.

[24] Lee T.W., Brownlee K.G., Conway S.P., Denton M., Littlewood J.M. (2003). Evaluation of a new definition for chronic Pseudomonas aeruginosa infection in cystic fibrosis patients. J. Cyst. Fibros..

[25] Bouka A., Tiede H., Liebich L., Dumitrascu R., Hecker C., Reichenberger F., Mayer K., Seeger W., Schulz R. (2012). Quality of life in clinically stable adult cystic fibrosis out-patients: Associations with daytime sleepiness and sleep quality. Respir. Med..

[26] Vandeleur M., Walter L.M., Armstrong D.S., Robinson P., Nixon G.M., Horne R.S.C. (2017). How Well Do Children with Cystic Fibrosis Sleep? An Actigraphic and Questionnaire-Based Study. J. Pediatr..

[27] Ohayon M.M., Carskadon M.A., Guilleminault C., Vitiello M.V. (2004). Meta-analysis of quantitative sleep parameters from childhood to old age in healthy individuals: Developing normative sleep values across the human lifespan. Sleep.

[28] Lopes-Pacheco M. (2019). CFTR Modulators: The Changing Face of Cystic Fibrosis in the Era of Precision Medicine. Front. Pharmacol..

[29] Heijerman H.G.M., McKone E.F., Downey D.G., Van Braeckel E., Rowe S.M., Tullis E., Mall M.A., Welter J.J., Ramsey B.W., McKee C.M. (2019). Efficacy and safety of the elexacaftor plus tezacaftor plus ivacaftor combination regimen in people with cystic fibrosis homozygous for the F508del mutation: A double-blind, randomised, phase 3 trial. Lancet.

[30] Middleton P.G., Mall M.A., Dřevínek P., Lands L.C., McKone E.F., Polineni D., Ramsey B.W., Taylor-Cousar J.L., Tullis E., Vermeulen F. (2019). Elexacaftor-Tezacaftor-Ivacaftor for Cystic Fibrosis with a Single Phe508del Allele. N. Engl. J. Med..

[31] Zhang L., Albon D., Jones M., Bruschwein H. (2022). Impact of elexacaftor/tezacaftor/ivacaftor on depression and anxiety in cystic fibrosis. Ther. Adv. Respir. Dis..

[32] Shakkottai A., Kim S., Mitchell R. (2023). 0799 Impact of Highly Effective Modulator Therapy on Obstructive Sleep Apnea in People with Cystic Fibrosis. Sleep.

