# Supplementary material for: Changes in Sleep in Children and Adults with Cystic Fibrosis and Primary Ciliary Dyskinesia over Time and after CFTR Modulator Therapy
Source: J Clin Med. 2023 Dec 11;12(24):7612. doi: 10.3390/jcm12247612 (PMC10744013; doi:10.3390/jcm12247612)
Supplement: Supplementary file 1 [file jcm-12-07612-s001.zip › jcm-2728708-supplementary.pdf]

Supplemental Materials - Changes in Sleep in Children and Adults with Cystic Fibrosis and Primary Ciliary Dyskinesia Over Time and After CFTR Modulator Therapy

Supplemental tables

Supplementary Table S1:

Correlation between changes in sleep, sleepiness, and quality of life over time in adults

		QOL emotional, change	QOL Physical, Change	QOL Social, Change	QOL Work, Change	QOL Total, Change
ESS, Change	Correlation Coefficient	0.073	0.184	-0.058	-0.152	-0.035
	Sig. (2-tailed)	0.708	0.339	0.764	0.441	0.855
	N	29	29	29	28	29
Global sleep quality, Change	Correlation Coefficient	0.128	-0.040	-0.144	0.081	0.201
	Sig. (2-tailed)	0.499	0.833	0.448	0.677	0.288
	N	30	30	30	29	30
Global Sleeping Score, Change	Correlation Coefficient	0.007	-0.224	0.180	0.117	0.122
	Sig. (2-tailed)	0.970	0.234	0.342	0.544	0.520
	N	30	30	30	29	30
	Correlation Coefficient	0.073	-0.060	0.322	-0.035	0.054

Sleep latency, Change	Sig. (2-tailed)	0.707	0.757	0.088	0.859	0.780
	N	29	29	29	28	29
Sleep disturbance, Change	Correlation Coefficient	0.093	0.334	**0.385***	0.224	**0.456***
	Sig. (2-tailed)	0.625	0.071	**0.036**	0.242	**0.011**
	N	30	30	30	29	30
Subjective Sleep score, Change	Correlation Coefficient	**0.458***	0.042	0.036	0.151	**0.402***
	Sig. (2-tailed)	**0.011**	0.824	0.852	0.434	**0.028**
	N	30	30	30	29	30

Supplementary Table S2:

Correlation between changes in sleep, sleepiness, QOL and FEV1, FEV1 change in adults

		FEV1, current	FEV1, Change
ESS, Change	Correlation Coefficient	0.164	0.199
	Sig. (2-tailed)	0.379	0.283
	N	31	31
Global Sleep quality, Change	Correlation Coefficient	-0.210	-0.001
	Sig. (2-tailed)	0.249	0.995
	N	32	32

Global Sleeping Score, Change	Correlation Coefficient	-0.213	-0.141
	Sig. (2-tailed)	0.242	0.443
	N	32	32
Sleep latency, Change	Correlation Coefficient	0.013	0.030
	Sig. (2-tailed)	0.943	0.875
	N	31	31
Sleep disturbance, Change	Correlation Coefficient	-0.150	0.198
	Sig. (2-tailed)	0.412	0.278
	N	32	32
Subjective Sleep, Change	Correlation Coefficient	0.075	-0.033
	Sig. (2-tailed)	0.682	0.860
	N	32	32
QOL emotional, Change	Correlation Coefficient	-0.150	-0.258
	Sig. (2-tailed)	0.437	0.176
	N	29	29
QOL Physical, Change	Correlation Coefficient	-0.252	0.208
	Sig. (2-tailed)	0.187	0.279
	N	29	29
QOL Social, Change	Correlation Coefficient	0.024	0.333

	Sig. (2-tailed)	0.904	0.078
	N	29	29
QOL Work, Change	Correlation Coefficient	-0.242	0.223
	Sig. (2-tailed)	0.215	0.254
	N	28	28
QOL Total, Change	Correlation Coefficient	-0.353	0.273
	Sig. (2-tailed)	0.061	0.152
	N	29	29

Supplementary Table S3:

Correlation between changes in sleep, sleepiness, and quality of life over time in pediatric patients

		Arousal disorder, change	ESS, change	Excessive Somnolence disorder, Change	Global Sleeping quality, Change	Hyperhidrosis disorder, Change	Initiating and Maintaining sleep disorder, Change	Sleep breathing disorder, Change	Sleep/wake transition disorder, Change
QOL emotional, Change	Correlation Coefficient	-0.068	0.246	0.360	0.063	0.275	0.337	0.111	0.259
	Sig. (2-tailed)	0.731	0.190	0.060	0.747	0.157	0.074	0.581	0.183
	N	28	30	28	29	28	29	27	28
QOL Physical, Change	Correlation Coefficient	-0.076	**.546****	**.402***	0.236	0.208	0.341	0.375	**.389***
	Sig. (2-tailed)	0.702	**0.002**	**0.034**	0.217	0.287	0.070	0.054	**0.041**
	N	28	30	28	29	28	29	27	28
QOL Social, Change	Correlation Coefficient	-0.154	0.297	0.182	0.073	0.044	0.091	0.246	0.122
	Sig. (2-tailed)	0.434	0.111	0.354	0.705	0.825	0.637	0.216	0.538
	N	28	30	28	29	28	29	27	28
QOL Total, Change	Correlation Coefficient	-0.117	**.497****	0.329	0.232	0.189	0.301	0.318	0.365
	Sig. (2-tailed)	0.546	**0.005**	0.082	0.218	0.325	0.106	0.099	0.051
	N	29	30	29	30	29	30	28	29
QOL Work, Change	Correlation Coefficient	0.125	0.251	0.333	0.198	0.010	0.058	0.370	**.461***
	Sig. (2-tailed)	0.588	0.248	0.140	0.378	0.966	0.796	0.108	**0.035**
	N	21	23	21	22	21	22	20	21

Supplementary Table S4:

Correlation between changes in sleep, sleepiness, QOL and FEV1, FEV1 change in pediatric patients

		FEV1, current	FEV1, change
Global Sleeping Quality, Change	Correlation Coefficient	-0.093	0.007
	Sig. (2-tailed)	0.637	0.974
	N	29	25
Hyperhidrosis disorder, Change	Correlation Coefficient	-0.181	-0.378
	Sig. (2-tailed)	0.365	0.068
	N	27	24
Initiating and Maintaining sleep disorder, Change	Correlation Coefficient	-0.033	0.015
	Sig. (2-tailed)	0.863	0.940
	N	29	26
Sleep breathing disorder, Change	Correlation Coefficient	-0.021	-0.336
	Sig. (2-tailed)	0.918	0.117
	N	26	23
Sleep/wake transition disorder, Change	Correlation Coefficient	0.114	0.018
	Sig. (2-tailed)	0.572	0.933
	N	29	23

Arousal disorder, Change	Correlation Coefficient	-0.024	0.210
	Sig. (2-tailed)	0.905	0.324
	N	27	24
ESS Change	Correlation Coefficient	0.008	-0.141
	Sig. (2-tailed)	0.966	0.493
	N	29	26
Excessive Somnolence disorder, Change	Correlation Coefficient	-0.023	-0.150
	Sig. (2-tailed)	0.908	0.485
	N	27	24

Supplementary Table S5:

Changes over time in sleep, sleepiness and QOL characteristics in total group

	Total N=67			CF-PI N=37		CF-PS N=15		PCD N=15		
	Baseline	Follow up	P value	Baseline	Follow up	Baseline	Follow up	Baseline PCD	Follow up	P value PI, PS, PCD
Global sleep quality	85.8 (79.3-95.2)	85.7 (71.4-90.7)	**0.011**	85.7 (80.8-91.4)	85.7 (72.6-92.1)	90.5 (76.8-95.7)	85.7 (71.4-90.7)	86 (76.2-96.1)	80.9 (74.4-89.3)	0.517, **0.006,** 0.084

ESS	83.3 (70.8-90.6)	83.3 (71.4-91.6)	0.669	76.6 (70.8-87.5)	87.5 (80.7-92.5)	87.5 (74.0-100)	75 (66.6-90)	83.3 (75-87.5)	73.3 (62.5-90)	0.081, 0.102, 0.209
QOL Total,	77.2 (65.5-89.1)	76.6 (64.0-89.2)	0.980	77.9 (71.2-93.5)	84.5 (75-90.1)	66.9 (52.6-85.7)	75 (61.9-84.7)	78.2 (60.9-86.5)	64 (52.4-77.5)	0.830, 0.245, 0.096

Supplementary Table S6:

Correlation between changes in sleep, sleepiness and QOL in total group

		ESS, Change	Global sleep quality, Change	Global Sleeping Score, Change
QOL emotional, Change	Correlation Coefficient	0.111	0.088	-0.225
	Sig. (2-tailed)	0.403	0.509	0.086
	N	59	59	59
QOL Physical, Change	Correlation Coefficient	**0.306**[*]	0.088	-0.225
	Sig. (2-tailed)	**0.018**	0.505	0.086
	N	59	59	59
QOL Social, Change	Correlation Coefficient	0.103	-0.039	-0.123
	Sig. (2-tailed)	0.440	0.768	0.354

	N	59	59	59
QOL Work, Change	Correlation Coefficient	-0.016	0.157	-0.122
	Sig. (2-tailed)	0.909	0.270	0.393
	N	51	51	51
QOL Total, Change	Correlation Coefficient	0.230	0.188	-0.156
	Sig. (2-tailed)	0.079	0.149	0.235
	N	59	60	60

Supplementary Table S7:

Changes in sleep, sleepiness and QOL in patients off modulators

Pediatric patients	Baseline	Follow up	P value pediatric patients	Adult patients	Baseline	Follow up	P value adult patients
**SDSC**				**PSQI**			
Global Sleep quality, %	82.7 (72.1-96.0)	88 (70.7-92.3)	0.420	Global Sleep quality	85.7 (78.6-88.3)	80.9 (71.4-87.3)	**0.021**
Arousal disorder, %	100 (100-100)	100 (100-100)	0.138	Sleep latency	100 (75-100)	83.3 (66.6-100)	**0.034**
Excessive Somnolence disorder, %	70 (65-95)	84 (68-92)	0.210	Sleep disturbance	87.5 (79-91.7)	79.2 (64.6-86.6)	**0.019**

Hyperhidrosis disorder, %	100 (62.5-100)	100 (60-100)	0.332	Subjective sleep	100 (66.6-100)	100 (66.6-100)	0.721
Initiating and Maintaining sleep disorder, %	81 (60.7-92.8)	77.1 (68-82.8)	0.472	Sleep duration	6.5 (6-7.3)	6.5 (5-7.3)	0.37
Sleep breathing disorder, %	91.7 (73-100)	93.3 (66.6-100)	0.878				
Sleep/wake transition disorder, %	91.7 (79.2-100)	93.3 (86.6-100)	0.660				
**ESS**				**ESS**			
ESS, %	75 (66.6-86.6)	86.7 (70.8-92.5)	0.214	ESS	83.3 (75-87.5)	87.5 (70.8-92.5)	0.938
**PedOL**				**QOL-B**			
QOL Total, %	79.9 (71.3-84.5)	79.9 (63.5-88.6)	0.538	QOL Total	76.8 (65.0-87.6)	75.7 (61.3-78.5)	0.140

Supplementary Table S8:

Changes in sleep, sleepiness and QOL in patients on modulators

Pediatric patients	Baseline	Follow up	P value pediatric patients	Adult patients	Baseline	Follow up	P value adult patients
**SDSC**				**PSQI**			

Global sleep quality, %	94.2 (86.6-97.2)	91.8 (87.3-96.5)	0.530	Global Sleep quality	85.8 (76.2-92.9)	76.2 (71.4-85.8)	0.078
Arousal disorder, %	100 (100-100)	100 (95-100)	0.705	Sleep latency	100 (87.5-100)	83.3 (54.2-100)	0.082
Excessive Somnolence disorder, %	95 (85-100)	88 (81-100)	0.260	Sleep disturbance	79.2 (70.8-95.8)	87.5 (67.7-93.7)	0.468
Hyperhidrosis disorder, %	100 (100-100)	100 (100-100)	0.276	Subjective sleep	100 (66.6-100)	100 (66.6-100)	0.717
Initiating and Maintaining sleep disorder, %	89.3 (83.5-94.8)	83.3 (80-90)	**0.054**	Sleep duration	6.5 (6-7)	6 (5.5-7)	0.61
Sleep breathing disorder, %	100 (93.8-100)	100 (83.3-100)	0.068				
Sleep/wake transition disorder, %	100 (83.3-100)	96.6 (93.3-100)	0.889				
**ESS**				**ESS**			
ESS, %	89.1 (71.9-100)	90 (76.7-93.3)	0.784	ESS	87.1 (75.4-95.8)	75 (68.7-83.3)	0.083
**PedsOL**				**QOL-B**			
QOL Total, %	93.0 (56.4-94.5)	82.6 (66.8-96.4)	0.875	QOL Total,	71.2 (53.5-81.1)	79.6 (64.6-91.8)	**0.016**
